# Autoimmunity against p53 predicts invasive cancer with poor survival in patients with an ovarian mass

**DOI:** 10.1054/bjoc.2000.1446

**Published:** 2000-10-26

**Authors:** F D Vogl, M Frey, R Kreienberg, I B Runnebaum

**Affiliations:** Department of Obstetrics and Gynaecology, University of Ulm, Ulm, 89075, Germany; Deutsches Krebsforschungszentrum, Steinbeis Transfer Zentrum, Heidelberg, 69120, Germany; Department of Obstetrics and Gynaecology, University of Freiburg, Hugstetterstrasse 55, Freiburg, 79106, Germany; Unit of Genetic Epidemiology, International Agency for Research on Cancer, Lyon Cedex 08, 69372, France

**Keywords:** p53, cohort study, adjusted analysis, prognosis, ovarian cancer

## Abstract

Serum autoantibodies against the p53 protein (p53 AAb) were analysed with a newly developed enzyme-linked immunosorbent assay (ELISA) based on highly purified and renatured p53. In a hospital-based cohort study, preoperative sera from 113 patients with ovarian cancer, 15 patients with borderline tumours and 117 patients with benign tumours of the ovaries were studied. The prevalence of p53 AAb in patients with invasive cancer was 19% (21/113). No p53 AAb were found in patients with borderline lesions or benign tumours. The ELISA had a specificity for malignancy of 99% (1 of 117; false-positive from a patient with severe diabetes mellitus) and a likelihood ratio (LR+) for a positive test result of 21.7 (elevated CA125 and malignancy: LR+ 3.7). p53 AAb were only detectable in patients with immunohistochemical staining of nuclear p53 in the tumour (*P*= 0.006). Presence of p53 AAb positively correlated with tumour stage (*P*= 0.034) and grade (*P*= 0.009). Kaplan–Meier analysis showed both a shortened overall survival (*P*= 0.0016, log-rank) and relapse-free survival (*P*= 0.055) for p53 AAb-positive patients (median follow-up 22 months). High titres related to even worse prognosis. p53 AAb independently related to poor survival adjusting for stage (*P*= 0.026), grade (*P*= 0.029) and residual disease after surgery (*P*= 0.005). Preoperative findings of adnexal mass with serum p53 AAb are strongly suggestive of an aggressive invasive ovarian cancer. © 2000 Cancer Research Campaign


*British Journal of Cancer* (2000) **83**(10), 1338–1343
© 2000 Cancer Research Campaign
doi: 10.1054/ bjoc.2000.1446, available online at http://www.idealibrary.com on IDEAL®

### Autoimmunity against p53 predicts invasive cancer with poor survival in patients with an ovarian mass

**FD Vogl[1,4], M Frey[2], R Kreienberg[1] and IB Runnebaum[3]**

[1]Department of Obstetrics and Gynaecology, University of Ulm, 89075 Ulm, Germany; [2]Deutsches Krebsforschungszentrum, Steinbeis Transfer Zentrum, 69120 Heidelberg, Germany; [3]Department of Obstetrics and Gynaecology, University of Freiburg, Hugstetterstrasse 55, 79106 Freiburg, Germany; [4]Current address: Unit of Genetic Epidemiology, International Agency for Research on Cancer, 69372 Lyon Cedex 08, France

**Summary** Serum autoantibodies against the p53 protein (p53 AAb) were analysed with a newly developed enzyme-linked immunosorbent assay (ELISA) based on highly purified and renatured p53. In a hospital-based cohort study, preoperative sera from 113 patients with ovarian cancer, 15 patients with borderline tumours and 117 patients with benign tumours of the ovaries were studied. The prevalence of p53 AAb in patients with invasive cancer was 19% (21/113). No p53 AAb were found in patients with borderline lesions or benign tumours. The ELISA had a specificity for malignancy of 99% (1 of 117; false-positive from a patient with severe diabetes mellitus) and a likelihood ratio (LR+) for a positive test result of 21.7 (elevated CA125 and malignancy: LR+ 3.7). p53 AAb were only detectable in patients with immunohistochemical staining of nuclear p53 in the tumour ($P$ = 0.006). Presence of p53 AAb positively correlated with tumour stage ($P$ = 0.034) and grade ($P$ = 0.009). Kaplan–Meier analysis showed both a shortened overall survival ($P$ = 0.0016, log-rank) and relapse-free survival ($P$ = 0.055) for p53 AAb-positive patients (median follow-up 22 months). High titres related to even worse prognosis. p53 AAb independently related to poor survival adjusting for stage ($P$ = 0.026), grade ($P$ = 0.029) and residual disease after surgery ($P$ = 0.005). Preoperative findings of adnexal mass with serum p53 AAb are strongly suggestive of an aggressive invasive ovarian cancer. © 2000 Cancer Research Campaign

**Keywords:** p53; cohort study; adjusted analysis; prognosis; ovarian cancer

Mutations of the p53 tumour suppressor gene are associated with tumorigenesis of most types of human cancers including ovarian cancer (Hollstein et al, 1991; Harris and Hollstein, 1993; Wen et al, 1999). Mutational inactivation of the p53 gene leading to an altered p53 protein occurs during tumorigenesis of approximately 50% of ovarian cancers (Marks et al, 1991; Milner et al, 1993; Wen et al, 1999). These alterations represent in more than 90% point missense mutations in the highly conserved core region resulting in a functionally inactive mutant protein (Soussi and May, 1996). The mutation frequently causes a delayed turnover of the protein and thereby an extended half-life compared to the wild-type protein. It accumulates in the tumour cell nucleus to high levels detectable by immunohistochemical methods (Lane, 1992; Legros et al, 1994; Runnebaum et al, 1996). Mutant p53 protein was shown to be immunogenic in different species leading to generation of autoantibodies (p53 AAb). These antibodies were directed against the immunodominant linear epitopes located in the amino- and carboxy-terminal regions of p53 (Schlichtholz et al, 1992; 1994; Lubin et al, 1993; Legros et al, 1994; Soussi and May, 1996).

p53 AAb were first detected in patients with breast cancer (Crawford et al, 1982), and in children with a variety of malignancies (Caron de Fromentel et al, 1987). Ovarian cancers were among the most immunogenic malignancies inducing p53 AAb response. In a comparative study, patients with cancer of the lung, breast, ovary or colon showed the highest p53 AAb prevalence (Angelopoulou et al, 1994).

Early investigations used electrophoresis-based systems such as Western blot analysis or immunoprecipitation applicable to only small patient samples (Crawford et al, 1982; Caron de Fromentel et al, 1987). The development of ELISA systems allowed for rapid and facilitated analysis of larger patient series, particularly to study the diagnostic and prognostic value of p53 AAb (Lubin et al, 1995; Angelopoulou et al, 1997). However, it has been hypothesized that the specificity of the early assay systems may have been limited due to single-step purification of the antigen p53 allowing binding of unspecific antibodies to epitopes exposed by p53 protein denaturation or to fragments of other proteins.

In the present study, an ELISA was developed based on double-purified and renatured, recombinant human p53 protein (Nedbal et al, 1997). Using this new assay, we assessed the prevalence of p53 AAb in preoperative patients with benign, borderline or malignant adnexal mass and the relation to histopathological and clinical parameters, particularly the clinical outcome. Implications for clinical management of patients with suspicious adnexal mass are discussed.

### METHODS

### Patients and materials

In this retrospective cohort study, we included 245 Caucasian women, 128 of whom were newly diagnosed with ovarian malignancy. One-hundred and seventeen patients (age range: 17–94 years, median 49 years) who were operated for benign lesions of the ovaries served as controls. Patients were enrolled at the

*Received 15 March 2000*
*Revised 10 July 2000*
*Accepted 12 July 2000*

*Correspondence to:* IB Runnebaum

Department of Obstetrics and Gynaecology of the University of Ulm during the period between January 1993 and November 1997. Diagnoses were proven histologically. Cases and controls were included on the basis of availability of pre-treatment serum samples. Serum had been obtained by centrifugation and was stored at –80°C until analysis. One hundred and thirteen women aged between 21 years and 89 years (median 61 years) had primary invasive ovarian cancer. Fifteen patients (age range: 23–75 years, median 50 years) were diagnosed with borderline lesions of the ovaries. Patients with borderline tumours were initially treated by oophorectomy. Patients with invasive cancer underwent cytoreductive surgery followed by chemotherapy. They were postoperatively staged according to the classification of the International Federation of Gynaecologists and Obstetricians (FIGO). Where complete surgical cytoreduction could not be achieved, the presence of visible residual tumour was recorded. Forty-five patients had visible tumour residues, 40 patients were resected R0 (information missing on 28). Platinum-based chemotherapy was given to 76 of 113 (67%) of the patients. Thirteen patients with stage I disease received no adjuvant therapy. Eighteen patients with stage III or IV received chemotherapy not containing platinum. Three patients with stage II and stage III disease refused chemotherapy. Another three patients with progressive disease underwent surgery, but died due to poor general condition before chemotherapeutical treatment could be started. Clinical data were obtained from the patients' charts and the histopathological reports. Values of the tumour marker CA125 were routinely determined at admission. Values below 35 U $ml^{-1}$ were considered normal. CA125 test results were available for 106 cancer cases and 112 controls with benign lesions. Patients with invasive cancer were followed up with respect to relapse and survival until November 30, 1998. Patients with borderline lesions and those with benign tumours were screened only for the presence of serum p53 AAb and excluded from further analysis.

### ELISA

The ELISA was based on double-purified recombinant human wild-type p53 protein (Nedbal et al, 1997). In brief, p53 was expressed in *E. coli* tagged aminoterminally with $His_6$ and purified through metal chelate affinity chromatography under denaturing conditions (8 M urea). The denatured p53 protein was refolded by stepwise removal of urea in a dialysis procedure and further purified through gel permeation (Sephadex 200, Pharmacia, Erlangen, Germany). Integrity of refolded p53 was confirmed with the monoclonal antibody PAb 421 recognizing wild-type p53 binding sequence-specifically to a 20 bp oligodeoxyribonucleotide (5′ GGACATGCCCGGGCATGTCC-3′). Microtitre plates (Maxisorp, Nunc, Wiesbaden, Germany) were coated with 50 µl per well of double-purified p53 (0.2 µg $ml^{-1}$) in PBSI. Plates were incubated overnight at 4°C and blocked for 1 h at 37°C in 5% non-fat milk powder (Merck, Darmstadt, Germany) in PBS. Serum was diluted 1:100 and incubated for 1 h at 37°C. After washing with PBS/0.05% Tween 20, peroxidase-conjugated goat antiserum to human immunoglobulin (Dianova, Hamburg, Germany) was added and incubated at 37°C for 30 min. Bound antibody was detected with tetramethylbenzidine and the results monitored in an automatic microtitre plate reader. ELISA cut-off was defined as duplicate of the mean of the absorption using sera from 100 healthy blood donors without a history of cancer. Cut-off corresponded to an antibody titre of 15 binding units. For measuring the antibody titre the assay was standardized using a positive human serum (positive standard) at ample supply showing no change of titre during storage. Binding activity of 1:600 diluted positive standard was defined as 1 binding unit. The binding activity showed linearity over a broad range with 1 binding unit lying in the middle of the linear line. Internal standard curve was used for calculation of the corresponding binding units of the human sera tested. Positive sera were diluted in a range from 1:100 to 1:100 000 to keep the analysis in the linear range of the standard curve. All samples were assayed in duplicate and all quantitative values were means of duplicate determinations. The analyst was unaware of any clinical patient data.

### Western blot

For confirmation of ELISA test results Western blots were performed. The amount of purified p53 protein loaded on a minigel was 0.3 µg $lane^{-1}$, which converts into 5 µg $cm^{-2}$ on the membrane. The sera were tested in a dilution of 1:100. Bound antibodies were detected with alkaline phosphatase-coupled goat anti-human antibodies and colour developed using bromo-chloro-indolyl phosphate/nitroblue tetrazolium (BCIP/NBT).

### Immunohistochemistry of p53 protein in tumour tissue

Results of routine immunohistochemical (IHC) staining of p53 in tumour tissue were available from 56 patients. Tissue sections (7 µm) were taken from paraffin-embedded ovarian tumour specimens, mounted on glass slides, and stained by the avidin biotin immunoperoxidase method using the anti-human p53 mouse monoclonal antibody DO-1 (Dianova). Immunoreactivity was assessed recording proportion of stained cells and staining intensity. Percentages of positive cells were scored 1 for 0–10%, 2 for 10–50% and 3 for > 50%. Tissue sections containing 10% or more stained tumour cells were considered as IHC-positive. Evaluations were independently performed by two pathologists of the institution.

### Statistical analysis

For patients with invasive ovarian cancer, Fisher's exact test was applied to test for an association of p53 AAb with each of the following dichotomized study parameters: Age (< 50 years vs ≥ 50 years), menopausal status (premenopausal vs postmenopausal), oestrogen and progesterone receptor status (IRS ≤ 3 vs IRS > 3; immuno-reactive score, score for staining intensity × score for percentage of positive cells), tumour cell type (serous vs other than serous), and presence of lymph-node metastases at primary surgery (present vs absent). In some cases information on particular parameters was not available.

Cochran-Armitage trend test was conducted across the polytomous variables tumour stage (FIGO), grading, and immunohistochemical results on p53. Our hypothesis was that the frequency of p53 AAb would be higher in patients with advanced tumour stages or less differentiated tumours. We also expected that the immune response to p53 would depend on the grade of p53 overexpression in tumour cells as assessed by the proportion of IHC-stained cells and staining intensity. Therefore *P*-values of the trend test were calculated one-sided.

*© 2000 Cancer Research Campaign* 

Person-months were accumulated up to date of relapse and of death, loss to follow-up or end of the follow-up period. Patients were stratified by presence or absence of p53 AAb. Kaplan–Meier curves were calculated for relapse-free interval and overall survival time. Differences between the Kaplan–Meier curves were evaluated by the log-rank test. Separately, the p53 AAb titre was considered as a predictive variable for survival. Starting at median (424 units) and then cutting at each value above and below until significance was obtained, a cut-off value was empirically selected. The level of significance was set to 0.05. All data analyses were carried out using SAS software (SAS Institute Inc, Cary NC, USA).

### RESULTS

Descriptive characteristics of the 113 ovarian cancer patients evaluated in the study are shown in Table 1. The prevalence of p53 AAb as detected with the described ELISA in the ovarian cancer patients at the time of diagnosis was 18.6% (21 of 113). In these patients titres ranged from 20 units to 51 400 units. Median was 424 units. Two patients had excessively high titres of 23 600 units and 51 400 units. The specificity of our p53 AAb ELISA was 99.2%. The assay showed a positive reaction only for one out of 117 patients in the control group, who had a titre of 34 units (mean of three independent duplicate measurements). The histology diagnosis of this patient was a unilateral benign cystadenoma simplex. p53 immunohistochemical analysis specifically performed on this material was negative. This patient with a severe type 1 diabetes mellitus was followed-up for 3 years without evidence of malignancy. Positivity for p53 AAb in the ELISA was confirmed by Western blot. All sera with an antibody titre higher than 30 units were clearly detectable in Western blot analysis. Some sera with a lower titre produced only a faint signal in Western blot analysis, possibly due to a lower sensitivity of this assay. None of the 15 patients with borderline lesions had serum p53 AAb detectable by ELISA or Western blot analysis. In terms of its use as a diagnostic test for malignancy, the likelihood ratio for a positive test result in the ELISA was 21.7; the likelihood ratio for a negative result was 0.8. For comparison, the sensitivity of the CA125 test was 84.9% with specificity 76.8%, likelihood ratios were 3.7 for a positive result and 0.2 for a negative result.

Table 1 shows the presence of p53 AAb in relation to clinicopathological parameters. In patients with FIGO stage I and II disease, three of 36 (8%) had p53 AAb in their serum. The frequency of p53 AAb was positively related with tumour stage, showing a higher prevalence in the advanced stages FIGO III and IV (14 of 62, 23% and four of 15, 27%, respectively; one-sided trend test over all stages: $P = 0.034$). Likewise, presence of p53 AAb was related with grade of tumour cell differentiation (one-sided trend test: $P = 0.009$). The presence of p53 AAb was independent of the menopausal status of the patient and immunohistochemically determined hormone receptor status. p53 AAb were not associated with particular histologic cell types of the tumour nor lymph-node involvement.

Patients were followed-up for a median time of 22 months (range: 1–68 months). Mortality during follow-up was significantly higher in the p53 AAb-positive group than in the p53 AAb-negative group (71% vs 32%, $P = 0.001$). Kaplan–Meier analysis revealed that patients with p53 AAb died significantly earlier (Figure 1, $P = 0.002$). The curves were nearly identical during the first 12 months of follow-up. One year survival rates were 85% and 84%, respectively. Thereafter, the graph for the p53 AAb-positive group decreased rapidly. Survival rates at 24 months were 68% and 39%, survival rates at 36 months were 61% and 19% for the AAb-negative and the AAb-positive group, respectively.

**Table 1** Characteristics of ovarian cancer patients and association of p53 AAb with clinicopathological parameters (Fisher's exact test). Given is the number and percentage of patients with positive result in the ELISA relative to the total number

| Feature | *n* | % | *P*-value |
|---|---|---|---|
| Age (years) | | | |
| < 50 | 2/26 | 8 | |
| ≥ 50 | 19/87 | 22 | 0.151 |
| Menopausal status | | | |
| premenopausal | 2/23 | 9 | |
| postmenopausal | 15/73 | 21 | 0.346 |
| Oestrogen receptor: | | | |
| negative (IRS ≤ 3) | 13/70 | 19 | |
| positive (IRS > 3) | 4/24 | 17 | 1.000 |
| Progesterone receptor | | | |
| negative (IRS ≤ 3) | 14/77 | 18 | |
| positive (IRS > 3) | 3/17 | 18 | 1.000 |
| Stage (FIGO) | | | |
| I | 2/23 | 9 | |
| II | 1/13 | 8 | |
| III | 14/62 | 23 | |
| IV | 4/15 | 27 | 0.034[a] |
| Grading | | | |
| 1 | 1/13 | 8 | |
| 2 | 4/41 | 10 | |
| 3 | 16/57 | 28 | 0.009[a] |
| Tumour cell type | | | |
| serous | 12/68 | 18 | |
| other than serous | 9/45 | 20 | 1.000 |
| Lymph-node metastasis | | | |
| yes | 4/26 | 15 | |
| no | 12/74 | 16 | 1.000 |
| Relapse | | | |
| No | 9/61 | 15 | |
| Yes | 12/49 | 24 | 0.228 |
| Death | | | |
| No | 6/68 | 9 | |
| Yes | 15/44 | 34 | 0.001 |

[a]Trend test, one-sided *P*-value

We separately studied variables previously identified to be predictors of poor outcome in ovarian cancer (stage of disease, tumour grading, residual tumour after surgery). Log-rank test confirmed for our cohort that patients were at higher risk of earlier death when having advanced FIGO III and IV stage ($P = 0.0002$), high tumour grading ($P = 0.0003$), or residual tumour after surgery ($P = 0.0007$). To investigate whether p53 AAb was an independent predictor, we repeated the analysis and adjusted for these variables. Survival was still significantly different by p53 AAb status after adjusting for tumour stage ($P = 0.026$), grading ($P = 0.029$) and presence or absence of residual tumour ($P = 0.005$). Differences in survival curves of p53 AAb-positive and p53 AAb-negative patients were also significant ($P = 0.030$) when only patients treated with platinum-based chemotherapy were considered (plots not shown).

Figure 2 shows the Kaplan–Meier curves with relapse as the end-point of follow-up. Patients with detectable p53 AAb had a shorter relapse-free survival time. The difference was of borderline significance ($P = 0.055$).

*© 2000 Cancer Research Campaign*

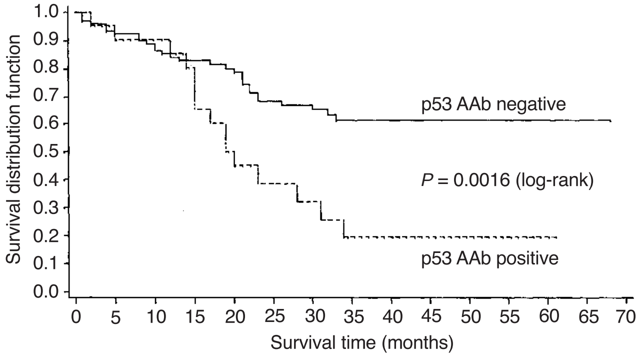

**Fig. 1:** Overall survival time in ovarian cancer patients by presence of p53AAb.

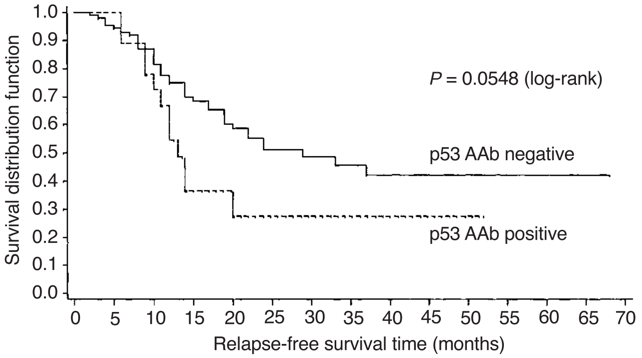

**Fig. 2**: Relapse-free survival time in ovarian cancer patients by presence of p53AAb.

**Table 2** Serum p53 AAb and p53 immunohistochemistry

| | p53 AAb status[b] | | |
|---|---|---|---|
| PP[a] | Positive | Negative | Total |
| 0–10% | 0 (0)[b] | 18 (100) | 18 |
| 10–50% | 2 (20) | 8 (80) | 10 |
| > 50% | 10 (36) | 18 (64) | 28 |
| Total | 12 (21) | 44 (79) | 56 |

[a]Proportion of positive-stained cells; [b]Number of patients (%);
Statistic: trend test ($P = 0.002$, one-sided)

Comparing survival in relation to antibody level within the p53 AAb-positive patients, a significant difference was obtained with the cut-off value set to 250 units, which corresponds to the lower tertil in that sample. Survival was shorter for patients whose p53 AAb titres lay above this value ($P = 0.027$). The median survival times in these two groups were 17 and 34 months, respectively.

p53 AAb were detected only in sera of patients with tumours containing 10% or more cells immunohistochemically positive for nuclear p53 overexpression ($P = 0.006$). Table 2 shows the frequencies of serum p53 AAb in relation to the proportion of immunohistochemically positive-stained cells. No p53 AAb were detectable when IHC was negative, whereas p53 AAb were present in 36% of patients whose tumours had more than half of the cells stained positive. The trend test confirmed our hypothesis of a positive association of p53 AAb frequency with the proportion of IHC-stained cells ($P = 0.002$). No association was found between p53 AAb and the staining intensity. p53 AAb were not detectable in 26 of 38 (68%) patients whose tumours showed p53 protein overexpression in tissue. Within this group, 18 of 28 (64%) of patients with more than 50% of cells positive for p53 by IHC had a negative antibody test.

### DISCUSSION

Based on our newly developed ELISA, we found p53 AAb in 21 of 113 (19%) ovarian cancer patients with a specificity of 99% for invasive ovarian cancer. All ELISA-positive samples were confirmed by positive Western blot analysis. The one false-positive sample was taken from a patient with severe diabetes mellitus possibly with an immunological cross-reactivity against various nuclear proteins including p53. Previously reported prevalences of p53 AAb in ovarian cancer ranged from 15% (Angelopoulou et al, 1994) to 46% (Vogl et al, 1999). The prevalence of 19% in our cohort of ovarian cancer patients is comparable to that of other epithelial cancers, particularly colorectal cancer with an antibody frequency between 16% and 23% (Angelopoulou et al, 1994; 1997), breast cancer with reported p53 AAb frequencies between 9% and 15% (Schlichtholz et al, 1992; Crawford et al, 1982) and lung cancer with frequencies between 8% and 24% (Lai et al, 1998, Schlichtholz, 1994).

Prevalences in previous reports appeared to depend on the type of assay used. In ovarian cancer, higher prevalences were found when ELISAs were used for detection (Green et al, 1995; Gadducci et al, 1996; 1998; Vogl et al, 1999). In these studies, ELISA results were not validated by other assay techniques and no control groups were tested, raising questions about the specificity of these assays. In a comparative analysis of three ELISA systems recently published by others, our solid-phase ELISA using double-purified and renatured wild-type p53 protein provided the highest diagnostic accuracy in correctly identifying cancer cases from a cohort of cases and controls (Rohayem et al, 1999). The other two test systems were a solid-phase ELISA using eucaryotically expressed wild-type p53 and a two-site sandwich ELISA using native p53 extracts from tumour cells.

Presence of p53 AAb positively correlated with tumour grading ($P = 0.009$) and stage ($P = 0.034$), indicating an aggressive behaviour of p53-AAb-inducing ovarian cancers. Tumour aggressiveness was also reflected by the shortened survival time and relapse-free period of antibody carriers. There are only few data on the prognostic value of p53 AAb in ovarian cancer. Angelopoulou et al found in bivariate analysis a significantly increased risk only for relapse (Angelopoulou et al, 1996). In multivariate analysis, however, p53 AAb was not an independent prognostic factor. Gadducci et al found that progression-free survival and overall survival of advanced (FIGO III and IV) ovarian cancer patients were not related to preoperative serum p53 AAb status (Gadducci

© 2000 Cancer Research Campaign 

et al, 1999). The data based on our newly developed ELISA clearly attribute a prognostic value to p53 AAb. Presence of p53 AAb was still an independent predictor of worse clinical outcome even after adjusting for the well-established prognostic parameters. The association of poor overall survival with p53 AAb as detected by our ELISA was unequivocally more clear-cut than the association with p53 mutation in the tumour and/or p53 IHC reported in previous studies including ours. Particularly in multivariate analyses, p53 IHC was not an independent predictor of poor survival (Eltabbakh et al, 1997; Wen et al, 1999). Quantitation of p53 AAb titre generated additional predictive information on patients' outcome. Our results favourably compare with studies on breast cancer and lung cancer showing a positive p53 AAb status to be an independent prognostic variable for poor overall survival (Peyrat et al, 1995; Lai et al, 1998).

Prognosis of patients with invasive ovarian cancer has changed little over the past two decades. Although early-stage ovarian cancer is highly curable, more than two thirds of the patients present with advanced disease with a poor survival rate.

Transvaginal sonography (TVS) – in general of low specificity – has been demonstrated to be a valuable screening technique with a relatively high sensitivity for early-stage ovarian cancer in high-risk populations of asymptomatic women (DePriest et al, 1997). Tumour marker CA125 determination (sensitivity 84.9%, specificity 76.8% in the present study) has failed to compensate for the lack of specificity of TVS. Owing to its high specificity (99%) for malignancy, the p53 AAb ELISA is suitable to be employed in a two-stage procedure as confirmatory test on individuals who had a positive or suspicious screening test. In this case, the high likelihood ratio for a positive result of 21.7 of the p53 AAb ELISA provokes a conclusive change from pre-test to post-test probability of malignancy. Future prospective studies should test whether p53 AAb ELISA can help to increase the diagnostic accuracy of ultrasound in order to avoid the delay of operation on ovarian cancer patients with low morphologic suspicion index. A positive p53 AAb test result in patients with adnexal mass should prompt referral to a centre with an infrastructure allowing explorative laparotomy. If laparoscopy is initially performed, availability of immediate and accurate pathologic diagnosis in such a centre is an important prerequisite for the treatment of these patients (Dottino et al, 1999).

Mechanisms leading to p53 immunogenicity still remain unexplained. Several studies consistently demonstrated that most antibodies preferentially target immunodominant linear epitopes contained in the amino (residues 1–95) and carboxy (residues 300–393) termini of p53. Both are exposed to the immune system due to their location on the surface of the protein, while the core domain prone to mutations is buried in the molecule (Schlichtholz et al, 1992; 1994; Legros et al, 1993; 1994; Lubin et al, 1993). These observations suggested that the specific immune response could be triggered by the level of nuclear p53 expression (Soussi and May, 1996). Our data on ovarian cancer support this hypothesis. All patients with circulating p53 AAb showed positive tumour immunostaining indicating p53 accumulation ($P = 0.006$). Such an association has also been reported for breast cancer (Mudenda et al, 1994) and colorectal cancer (Houbiers et al, 1995). Additionally, we found that the frequency of p53 AAb was positively related ($P = 0.002$) to the proportion of stained tumour cells, indicating an underlying quantitative effect on antibody induction. Only a subset of patients with IHC-positive tumours had a positive result in our ELISA, suggesting that p53 accumulation in ovarian cancer is not sufficient to induce a detectable humoral response.

In summary, p53 AAb, as detected by double-purified and refolded p53 antigen, were highly specific for malignancy in patients with ovarian mass and correlated with aggressive behaviour of the cancer. Presence of p53 AAb independently predicted poor clinical outcome, particularly above a cut-off value of 250 units. Quantitation of p53 AAb might contribute additional prognostic information. Further studies may prove the p53 AAb ELISA to be a valuable tool to preoperatively identify patients with aggressive ovarian tumours. p53 AAb testing could improve diagnostic accuracy of screening methods and consequently reduce stage at detection, decrease stage-specific mortality and could help in clinical decision-making on therapeutic regimens such as adjuvant p53 gene replacement therapy in addition to standard chemotherapy.

### ACKNOWLEDGEMENTS

The authors thank Dr Hanswalter Zentgraf, Deutsches Krebsforschungszentrum, Heidelberg, for providing us with the p53 protein. This study was supported in part by the Deutsche Forschungsgemeinschaft (RU476/3–1) and the Zentrum for klinische Forschung of the University of Freiburg (ZKF 1/C9) granted to IBR.

### REFERENCES

Angelopoulou K, Diamandis EP, Sutherland DJ, Kellen JA and Bunting PS (1994) Prevalence of serum antibodies against the p53 tumor suppressor gene protein in various cancers. *Int J Cancer* **58**: 480–487

Angelopoulou K, Rosen B, Stratis M, Yu H, Solomou M and Diamandis EP (1996) Circulating antibodies against p53 protein in patients with ovarian carcinoma. Correlation with clinicopathologic features and survival. *Cancer* **78**: 2146–2152

Angelopoulou K, Straits M and Diamandis EP (1997) Humoral immune response against p53 protein in patients with colorectal carcinoma. *Int J Cancer* **70**: 46–51

Caron de Fromentel C, May-Levin F, Mouriesse H, Lemerle J, Chandrasekaran K and May P (1987) Presence of circulating antibodies against cellular protein p53 in a notable proportion of children with B-cell lymphoma. *Int J Cancer* **39**: 185–189

Crawford LV, Pim DC and Bulbrook RD (1982) Detection of antibodies against the cellular protein p53 in sera from patients with breast cancer. *Int J Cancer* **30**: 403–408

DePriest PD, Gallion HH, Pavlik EJ, Kryscio RJ and van Nagell JR, Jr. (1997) Transvaginal sonography as a screening method for the detection of early ovarian cancer. *Gynecol Oncol* **65**: 408–414

Dottino PR, Levine DA, Ripley DL and Cohen CJ (1999) Laparoscopic management of adnexal masses in premenopausal and postmenopausal women. *Obstet Gynecol* **93**: 223–228

Eltabbakh GH, Belinson JL, Kennedy AW, Biscotti CV, Casey G, Tubbs RR and Blumenson LE (1997) p53 overexpression is not an independent prognostic factor for patients with primary ovarian epithelial cancer. *Cancer* **80**: 892–898

Gadducci A, Ferdeghini M, Buttitta F, Fanucchi A, Annicchiarico C, Prontera C, Bevilacqua G and Genazzani AR (1996) Preoperative serum antibodies against the p53 protein in patients with ovarian and endometrial cancer. *Anticancer Res* **16**: 3519–3523

Gadducci A, Ferdeghini M, Buttitta F, Cosio S, Fanucchi A, Annicchiarico C and Genazzani AR (1998) Serum anti-p53 antibodies in the follow-up of patients with advanced ovarian carcinoma. *Anticancer Res* **18**: 3763–3765

Gadducci A, Ferdeghini M, Buttitta F, Cosio S, Fanucchi A, Annicchiarico C, Gagetti O, Bevilacqua G and Genazzani AR (1999) Assessment of the prognostic relevance of serum anti-p53 antibodies in epithelial ovarian cancer. *Gynecol Oncol* **72**: 76–81

© 2000 Cancer Research Campaign

Green JA, Robertson LJ, Campbell IR and Jenkins J (1995) Expression of the p53 gene and presence of serum autoantibodies in ovarian cancer: correlation with differentiation. *Cancer Detect Prev* **19**: 151–155

Harris CC and Hollstein M (1993) Clinical implications of the p53 tumor-suppressor gene. *N Engl J Med* **329**: 1318–1327

Hollstein M, Sidransky D, Vogelstein B and Harris CC (1991) p53 mutations in human cancers. *Science* **253**: 49–53

Houbiers JG, van der Burg SH, van de Watering LM, Tollenaar RA, Brand A, van de Velde CJ and Melief CJ (1995) Antibodies against p53 are associated with poor prognosis of colorectal cancer. *Br J Cancer* **72**: 637–641

Lai CL, Tsai CM, Tsai TT, Kuo BI, Chang KT, Fu HT, Perng RP and Chen JY (1998) Presence of serum anti-p53 antibodies is associated with pleural effusion and poor prognosis in lung cancer patients. *Clin Cancer Res* **4**: 3025–3030

Lane DP (1992) Cancer. p53, guardian of the genome. *Nature* **358**: 15–16

Legros Y, Lacabanne V, d’Agay MF, Larsen CJ, Pla M and Soussi T (1993) Production of human p53 specific monoclonal antibodies and their use in immunohistochemical studies of tumor cells. *Bull Cancer* **80**: 102–110

Legros Y, Lafon C and Soussi T (1994) Linear antigenic sites defined by the B-cell response to human p53 are localized predominantly in the amino and carboxy-termini of the protein. *Oncogene* **9**: 2071–2076

Lubin R, Schlichtholz B, Bengoufa D, Zalcman G, Tredaniel J, Hirsch A, de Fromentel CC, Preudhomme C, Fenaux P, Fournier G, Mangin P, Laurent-Pvig P, Pelletier G, Schlumberger M, Desgrandchamps F, Le Duc A, Peyrat JP, Janin N, Bressac B and Soussi T (1993) Analysis of p53 antibodies in patients with various cancers define B-cell epitopes of human p53: distribution on primary structure and exposure on protein surface. *Cancer Res* **53**: 5872–5876

Lubin R, Schlichtholz B, Teillaud JL, Garay E, Bussel A and Wild CP (1995) p53 antibodies in patients with various types of cancer: assay, identification, and characterization. *Clin Cancer Res* **1**: 1463–1469

Marks JR, Davidoff AM, Kerns BJ, Humphrey PA, Pence JC, Dodge RK, Clarke Pearson DL, Iglehart JD, Bast RC Jr and Berchuck A (1991) Overexpression and mutation of p53 in epithelial ovarian cancer. *Cancer Res* **51**: 2979–2984

Milner BJ, Allan LA, Eccles DM, Kitchener HC, Leonard RC, Kelly KF, Parkin DE and Haites NE (1993) p53 mutation is a common genetic event in ovarian carcinoma. *Cancer Res* **53**: 2128–2132

Mudenda B, Green JA, Green B, Jenkins JR, Robertson L, Tarunina M and Leinster SJ (1994) The relationship between serum p53 autoantibodies and characteristics of human breast cancer. *Br J Cancer* **69**: 1115–1119

Nedbal W, Frey M, Willemann B, Zentgraf H and Sczakiel G (1997) Mechanistic insights into p53-promoted RNA-RNA annealing. *J Mol Biol* **266**: 677–687

Peyrat JP, Bonneterre J, Lubin R, Vanlemmens L, Fournier J and Soussi T (1995) Prognostic significance of circulating P53 antibodies in patients undergoing surgery for locoregional breast cancer. *Lancet* **345**: 621–622

Rohayem J, Conrad K, Zimmermann T and Frank KH (1999) Comparison of the diagnostic accuracy of three commercially available enzyme immunoassays for anti-p53 antibodies. *Clin Chem* **45**: 2014–2016

Runnebaum IB, Kieback DG, Mobus VJ, Tong XW and Kreienberg R (1996) Subcellular localization of accumulated p53 in ovarian cancer cells. *Gynecol Oncol* **61**: 266–271

Schlichtholz B, Legros Y, Gillet D, Gaillard C, Marty M, Lane D, Calvo F and Soussi T (1992) The immune response to p53 in breast cancer patients is directed against immunodominant epitopes unrelated to the mutational hot spot. *Cancer Res* **52**: 6380–6384

Schlichtholz B, Tredaniel J, Lubin R, Zalcman G, Hirsch A and Soussi T (1994) Analyses of p53 antibodies in sera of patients with lung carcinoma define immunodominant regions in the p53 protein. *Br J Cancer* **69**: 809–816

Soussi T and May P (1996) Structural aspects of the p53 protein in relation to gene evolution: a second look. *J Mol Biol* **260**: 623–637

Vogl FD, Stickeler E, Weyermann M, T Kohler, Grill H, Negri G, Kreienberg R and Runnebaum IB (1999) p53 autoantibodies in patients with primary ovarian cancer are associated with higher age, advanced stage and a higher proportion of p53-positive tumor cells. *Oncology* **57**: 324–329

Wen W-H, Reles A, Runnebaum IB, Sullivan-Halley J, Bernstein L, Jones LA, Kreienberg R, El-Naggar A, Felix JC and Press MF (1999) p53 mutations and expression in ovarian cancers: Correlation with overall survival. *Int J Gynecol Pathol* **18**: 29–41

*© 2000 Cancer Research Campaign*
